# A case of airway aluminosis with likely secondary pleuroparenchymal fibroelastosis

**DOI:** 10.1186/s40248-019-0177-4

**Published:** 2019-04-15

**Authors:** Yuki Yabuuchi, Hitomi Goto, Mizu Nonaka, Hiroaki Tachi, Tatsuya Akiyama, Naoki Arai, Hiroaki Ishikawa, Kentaro Hyodo, Kenji Nemoto, Yukiko Miura, Isano Hase, Shuji Oh-ishi, Kenji Hayashihara, Takefumi Saito, Tatsuya Chonan

**Affiliations:** 1 Department of Respiratory Medicine, National Hospital Organization, Ibaraki Higashi National Hospital, Ibaraki, Japan; 2 Department of Medicine, Nikko Memorial Hospital, Ibaraki, Japan

**Keywords:** Interstitial pneumonia, Occupational lung disease, Trans-bronchial lung biopsy, Elemental analysis

## Abstract

**Background:**

Excessive inhalation of aluminium powder occasionally results in upper lobe predominant lung fibrosis, which is similar to idiopathic pleuroparenchymal fibroelastosis (IPPFE) and has been suggested to be secondary PPFE.

**Case presentation:**

A 67-year-old man who had worked in an aluminum-processing factory for 50 years visited our hospital complaining of exertional dyspnea. Chest computed tomography (CT) showed bilateral dense sub-pleural consolidation in the upper and middle lung fields, which was consistent with IPPFE; however, the possibility of secondary PPFE associated with aluminosis was not ruled out. Considering the patient’s critical condition, trans-bronchial lung biopsy (TBLB) rather than surgical lung biopsy was performed, with elemental analysis of the biopsied specimen. Unfortunately, the specimen obtained by TBLB did not contain alveolar tissue; therefore, pathological diagnosis of PPFE was not possible. However, radiographic findings were highly suggestive of PPFE. On elemental analysis, excessive amounts of aluminum were detected in the bronchiolar walls, establishing a diagnosis of airway aluminosis with likely secondary PPFE resulting from aluminium exposure.

**Conclusions:**

TBLB with elemental analysis might be useful in differentiating idiopathic PPFE from secondary causes in dust inhalation related disease, such as aluminosis. This case indicated that inhalation of aluminium might cause secondary PPFE, with attention needing to be paid to avoid further exposure.

## Background

Pleuroparenchymal fibroelastosis (PPFE) is characterized radiologically by upper lobe predominant and pathologically unclassified interstitial pneumonia [1]. Most cases of PPFE have been classified as idiopathic; however, it occasionally occurs secondary to other conditions, including drug administration, infection, post-transplantation and exposure to dust [2]. In particular, there have been reports of PPFE-like lesions associated with dust exposure, such as asbestos, aluminium and aluminosilicate [3, 4].

In this report, we describe a putative case of secondary PPFE found in a worker exposed to aluminium dust, which was diagnosed with chest computed tomography (CT), trans-bronchial lung biopsy (TBLB) and elemental analysis of the biopsied specimen. This report provides new insights into secondary as well as idiopathic PPFE.

## Case presentation

**Fig. 1:**
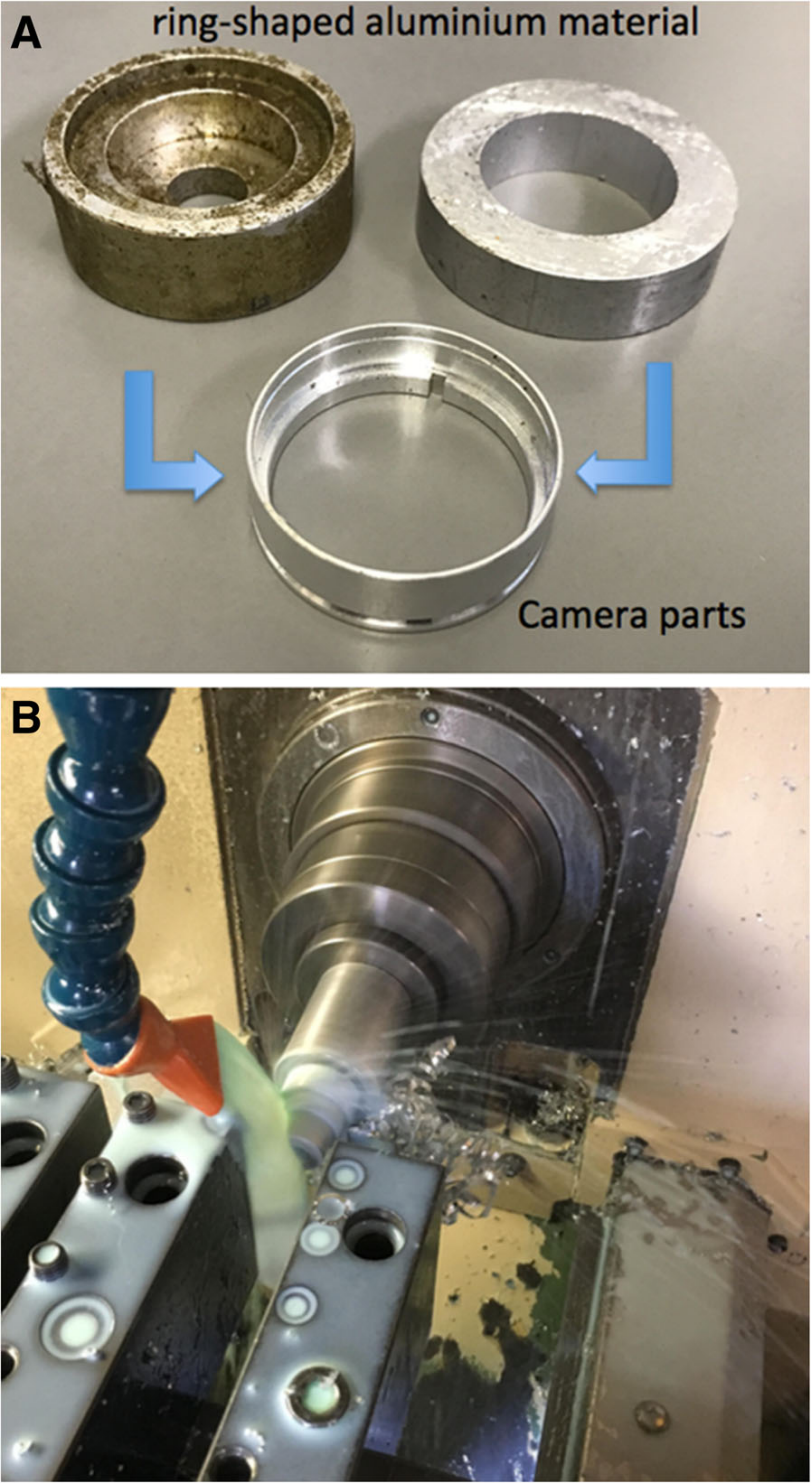
The patient was engaged in shaving ring-shaped aluminum material, such as the upper 2 images, and making camera parts, like the one below (**a**), with a curving machine (**b**). Workers are required to pour cutting oil on the material while cutting, as in (**b**), but our patient failed to do so, suggesting he might have inhaled a larger amount of aluminum-containing fumes than other worker in this occupation

The patient was a 67-year-old male non-smoker with an unremarkable past medical history. He was admitted to our hospital with a complaint of increasing dyspnea for 3 months. His parents managed a workshop in a small town, and he had worked in processing aluminum and brass to make camera parts since he was 18 years old. He did not wear a dust respirator and worked closely to the cutting machine (Fig. 1) because of his poor eyesight.

**Fig. 2:**
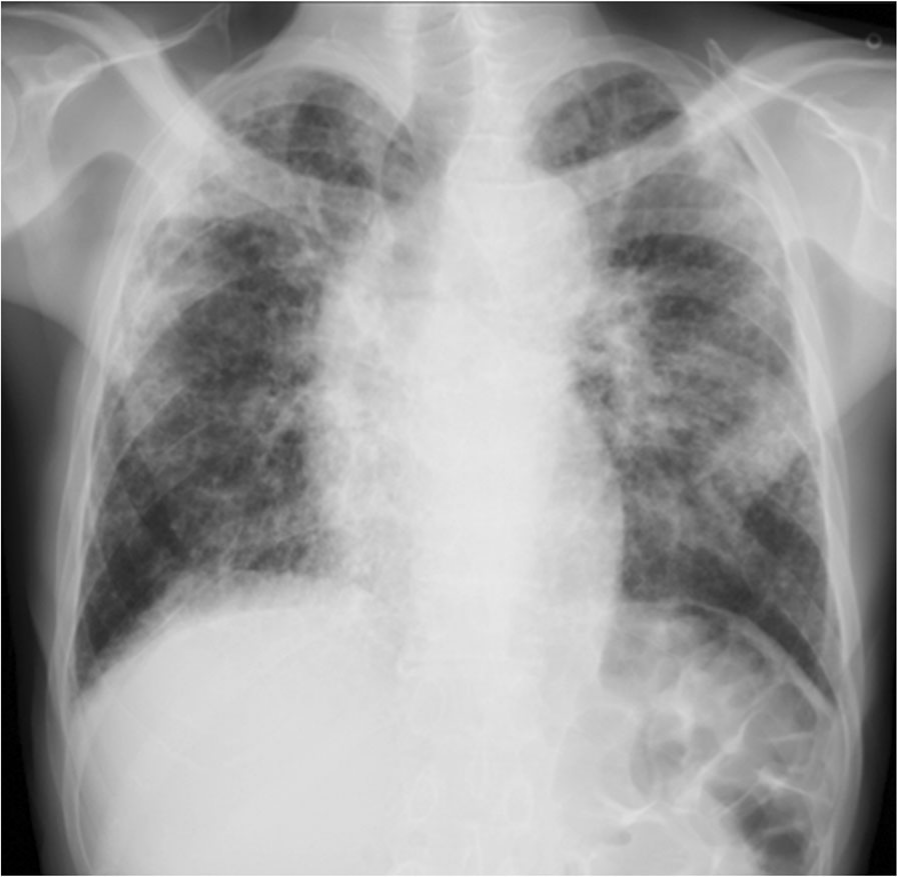
Chest X-ray showing bilateral pleural thickening in the upper and middle lung fields. The lung volume was reduced and reticulonodular shadows extended from the sub-pleura to deep inside the lungs, suggesting pulmonary fibrosis. The tracheal bifurcation was widened by traction of the upper lobes

**Fig. 3:**
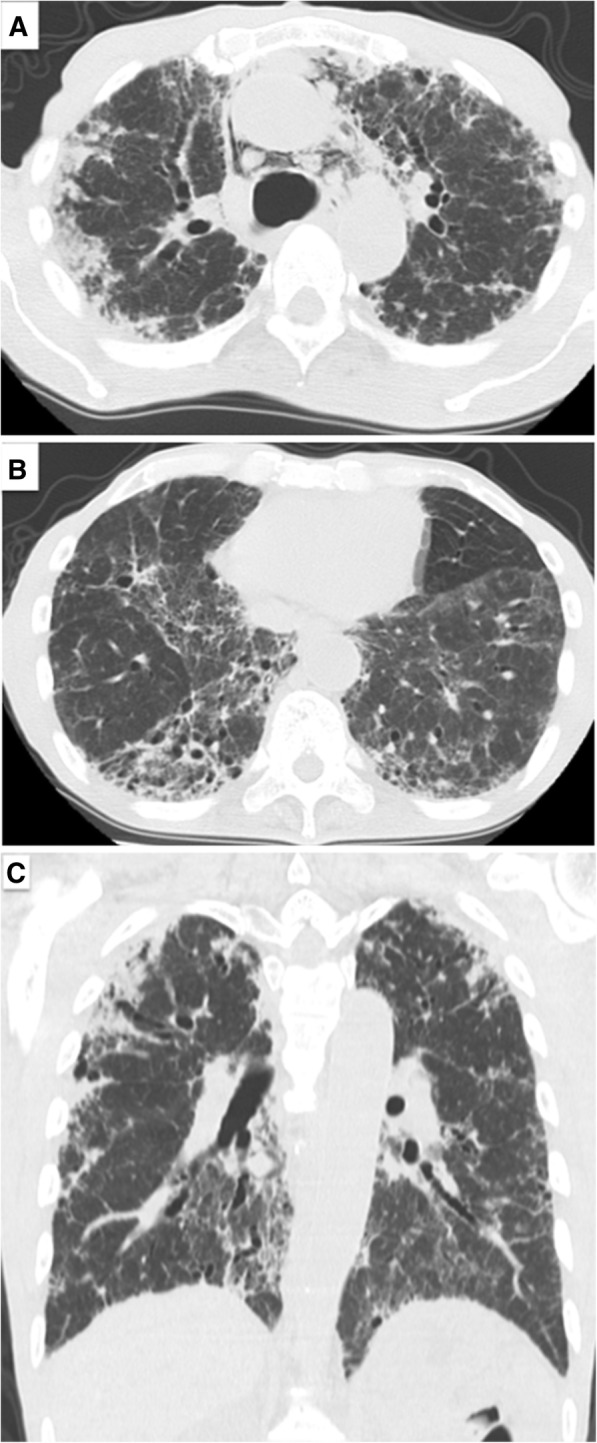
High-resolution computed tomography showing severe bilateral pleural thickening with shrunken and distorted upper lobes (**a**, **c**). There was mediastinal emphysema (**a**). On the other hand, reticulonodular shadows and bronchiectasis were distributed deep inside the lower lung (**b**, **c**)

On presentation, the patient was fully conscious with a height of 160 cm, a body weight of 47 kg, body temperature of 36.8 °C and percutaneous oxygen saturation (SpO_2_) of 94% on room air. Physical examination revealed fine crackles upon auscultation, especially on the bilateral upper lung areas. Otherwise, there were no abnormal physical findings. Laboratory data demonstrated elevated serum levels of Krebs von den Lungen-6 (KL-6; 1631 U/mL) and surfactant protein D (SP-D; 157.3 ng/mL), compared to normal values of < 500 U/mL and < 110 ng/mL, respectively. Antinuclear and autologous antibodies were all negative. Chest X-ray showed linear and reticulonodular shadows with marked bilateral pleural thickening in the upper lung fields (Fig. 2). Chest CT indicated bilateral dense sub-pleural consolidation, bronchiectasis and ground glass opacities in the bilateral upper lobes (Fig. 3). Pulmonary function tests showed a restrictive disorder: vital capacity (VC) of 1.12 L (34.6% of predicted), forced expiratory volume in 1.0 s (FEV_1_) of 0.99 L (42.5% of predicted), FEV_1_/FVC (forced vital capacity) of 74.4% and total lung capacity (TLC) of 2.48 L (47.7% of predicted). The patient was unable to perform the diffusion test correctly. TBLB was performed with bronchoscopy to get tissue samples of the right upper lobe (B2). Bronchoalveolar lavage fluid, obtained by infusing 150 mL of saline, revealed a total cell count of 6.0 × 10^4^/mL, comprised of 3% lymphocytes, 1% neutrophils, 2% eosinophils and 94% macrophages (Cluster of Differentiation, CD 4/8 ratio of 2.31), which was in the normal range. The biopsied specimen had fragments of bronchiolar wall with infiltration of lymphocytes in interstitial spaces; unfortunately, it did not contain alveolar tissue. A surgical diagnostic procedure was avoided due to the patient’s critical respiratory condition and instead, elemental analysis was performed on the existing specimen obtained by TBLB. The content of aluminum and other particles were measured in the TBLB sample.

**Fig. 4:**
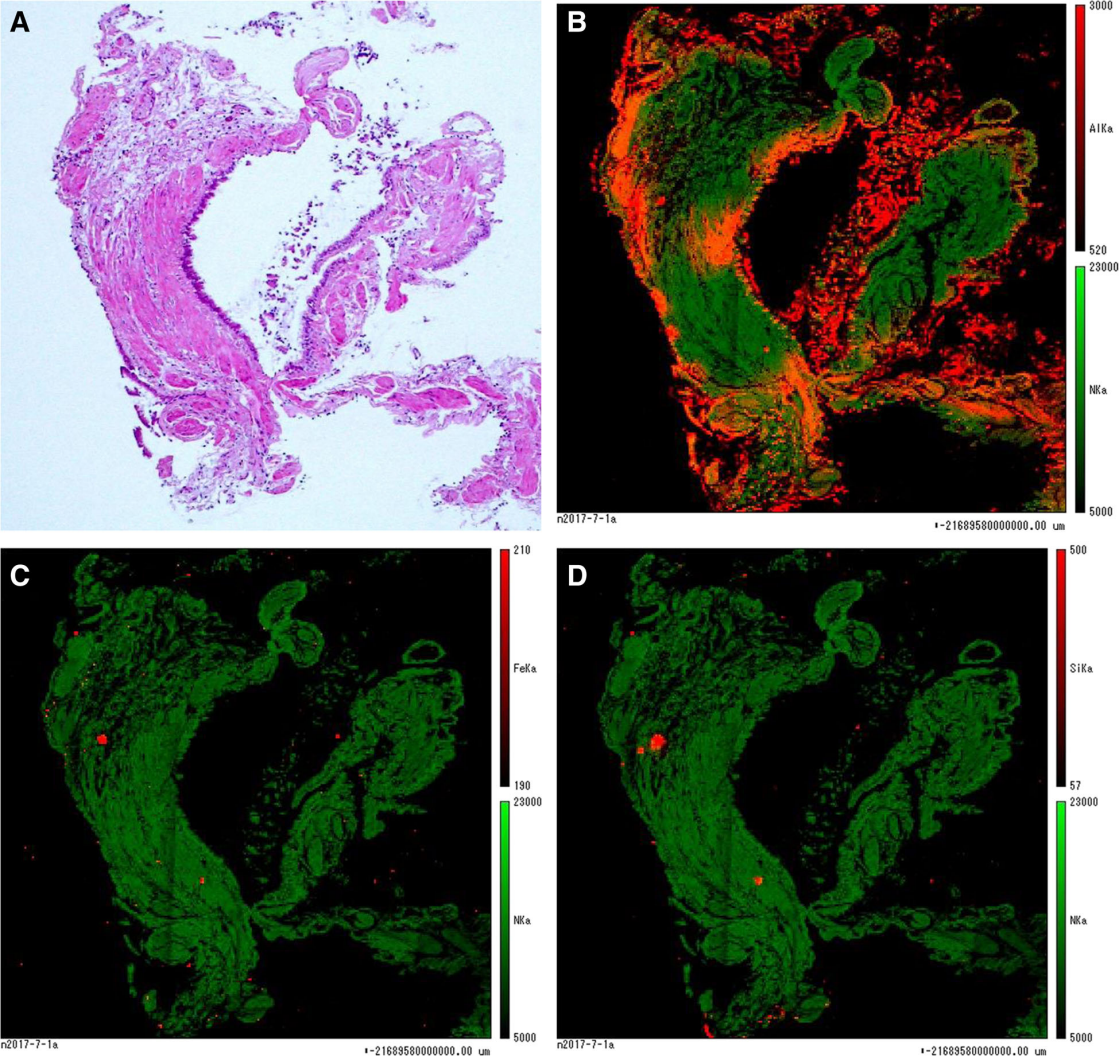
Elemental analysis of the biopsy specimen by electron probe X-ray microanalysis (EPMA). Aluminum was identified in a fragment of bronchial wall obtained by trans-bronchial lung biopsy (TBLB), after staining with hematoxylin and eosin (**a**). Deposition of elements in the specimen was shown by red to yellow colours. The green colour indicated deposition of nitrogen as a control. Significant amounts of aluminum were identified by EPMA as shown by red to yellow colours (**b**), whereas iron and silica were detected in much lower amounts compared with aluminum (**c, d**)

On elemental analysis of the biopsy specimen, a relatively high amount of aluminum was detected in comparison to trace amounts of other elements, including iron and silica (Fig.4). Based on the histological findings, the elemental analysis and the occupational history, the case was diagnosed as airway aluminosis. The patient’s condition was progressively getting worse; consequently, home oxygen therapy was implemented and the anti-fibrotic agent pirfenidone was administered. Six months later, a pneumothorax occurred and chest drainage was performed; however, his condition deteriorated, and he died of hypercapnic respiratory failure 1 month later. His family did not agree to an autopsy.

## Discussion

The etiology of PPFE has to be established yet; however, it has been suggested that dust inhalation is one of the causes of secondary PPFE [3, 4]. PPFE has been defined as upper lobe predominant sub-pleural fibrosis, with the diagnosis of PPFE needing radiological and pathological confirmation [1, 4].

In our case, the biopsied specimen contained only bronchiolar walls without alveolar tissue, and so did not allow histological confirmation of PPFE. Chest CT indicated upper lobe predominant pleural thickening with pulmonary fibrosis, bronchiectasis and pulmonary shrinkage, which was compatible with radiological findings for PPFE [1, 2]. On the other hand, there were features inconsistent with PPFE, including the presence of lower lobe as well as upper lobe fibrosis and lesions deep inside the lung were observed in addition to sub-pleural fibrosis. Furthermore, typical chest CT images of PPFE reveal a clear demarcation between the abnormal and normal parts of the lung [5], while it was ill-defined in the present case. It is controversial whether reticulonodular shadows in deep areas of the lung are an element of PPFE.

Pulmonary fibrosis caused by chronic inhalation of aluminum-containing dust or fumes is known as pulmonary aluminosis [6]. Radiologically, it is usually characterized by bilateral upper lung dominant reticulonodular or irregular shadows with pleural thickening, with rare exceptions of lower lung predominance, which is sometimes the case for PPFE-like lesions [4, 7]. Histological characteristics of pulmonary aluminosis are sub-pleural fibrosis with emphysematous lesions generated by fibrotic scarring [8]. Confirming elevated levels of aluminum in alveolar tissue is required to diagnose pulmonary aluminosis [8, 9].

In our case, increases in serum biomarkers KL-6 and SP-D, a restrictive change in pulmonary function and radiographic findings indicated that severe pulmonary fibrosis was involved. Since an invasive procedure like surgical lung biopsy (SLB) might have exacerbated the patient’s condition, we decided to only perform elemental analysis of the fragment of bronchiolar walls obtained by TBLB. This revealed an extensive deposition of aluminum, with small amounts of iron and silica, which was defined as airway aluminosis. This result indicated inhalation of large amounts of aluminium, which may have caused the PPFE-like lesions.

PPFE has a poor prognosis and effective therapies have not been identified. We administered the anti-fibrotic agent pirfenidone, which has been used for the treatment of idiopathic pulmonary fibrosis (IPF). There is no evidence on the efficacy of pirfenidone in fibrotic lung diseases other than IPF; however, a study evaluating its efficacy is ongoing. In our case, dyspnea was not improved for 6 months after introducing pirfenidone, when pneumothorax occurred and the patient died.

As in this case, it may be worth considering TBLB in cases of secondary PPFE-like lesions that are associated with dust exposure. Patients diagnosed as secondary PPFE with dust exposure might use the findings to obtain financial compensation.

## Conclusion

This case report described upper lobe predominant lung fibrosis in an aluminium welder. It indicated that excessive inhalation of alminium might cause secondary PPFE.

## Abbreviations

CD: Cluster of Differentiation
CT: Computed tomography
FEV_1_: Forced expiratory volume in 1.0 s
FVC: Forced vital capacity
IIPs: Idiopathic interstitial pneumonias
IPPFE: Idiopathic pleuroparenchymal fibroelastosis
KL-6: Krebs von den Lungen-6
SLB: Surgical lung biopsy
SP-D: Surfactant protein D
SpO_2_: Percutaneous oxygen saturation
TBLB: Trans-bronchial lung biopsy
TLC: Total lung capacity
VC: Vital capacity
